# Psychometric properties of the Press Ganey® Outpatient Medical Practice Survey

**DOI:** 10.1186/s12955-017-0610-3

**Published:** 2017-02-10

**Authors:** Angela P. Presson, Chong Zhang, Amir M. Abtahi, Jacob Kean, Man Hung, Andrew R. Tyser

**Affiliations:** 1 Department of Internal Medicine, Division of Epidemiology, University of Utah, Williams Building, 295 Chipeta Way, Salt Lake City, 84108 UT USA; 2 Department of Pediatrics, University of Utah, Salt Lake City, 84108 UT USA; 3 Department of Biostatistics, University of California, Los Angeles, 90095 CA USA; 4 Department of Orthopaedics, University of Utah, Salt Lake City, 84108 UT USA; 5 Department of Population Health Sciences, University of Utah, Salt Lake City, UT 84108 USA

**Keywords:** Consumer assessments, Patient satisfaction, Psychometrics, Press Ganey® medical practice survey, Confirmatory factor analysis

## Abstract

**Background:**

The Press Ganey® Medical Practice Survey (“Press Ganey® survey”) is a patient-reported questionnaire commonly used to measure patient satisfaction with outpatient health care in the United States. Our objective was to evaluate the reliability and validity of the Press Ganey® survey in a single institution setting.

**Methods:**

We analyzed surveys from 34,503 unique respondents seen by 624 providers from 47 specialties and 94 clinics at the University of Utah in 2013. The University of Utah is a health care system that provides primary through tertiary care for over 200 medical specialties. Surveys were administered online. The Press Ganey® survey consisted of 24 items organized into 6 scales: Access (4 items), Moving Through the Visit (2), Nurse Assistant (2), Care Provider (10), Personal Issues (4) and Overall Assessment (2). Missingness, ceiling and floor rates were summarized. Cronbach’s alpha was used to evaluate internal consistency reliability. Confirmatory factor analysis was used to assess convergent and discriminant validities.

**Results:**

Missingness was 0.01% for the total score and ranged from 0.8 to 11.4% across items. The ceiling rate was high at 29.3% for the total score, and ranged from 55.4 to 84.1% across items. Floor rates were 0.01% for the total score, and ranged from 0.1 to 2.1% across items. Internal consistency reliability ranged from 0.79 to 0.96, and item-scale correlations ranged from 0.49 to 0.9. Confirmatory factor analysis supported convergent and discriminant validities.

**Conclusion:**

The Press Ganey® survey demonstrated suitable psychometric properties for most metrics. However, the high ceiling rate can have a notable impact on quarterly percentile scores within our institution. Multi-institutional studies of the Press Ganey® survey are needed to inform administrative decision making and institution reimbursement decisions based on this survey.

## Background

Changes in the United States healthcare system have increasingly emphasized quality measurement, recognizing patient satisfaction with medical care as an important but somewhat controversial healthcare quality metric. Recently, the Medicare Provider Payment Modernization Act of 2014 (H.R.4015) ended the sustainable growth rate formula for determining physician reimbursement, supporting a reimbursement model that increasingly ties reimbursement to health care quality measures such as patient satisfaction [1–4].

Patient satisfaction has most prominently been measured by the standardized and nationally implemented Hospital Consumer Assessment of Healthcare Providers and Systems (H-CAHPS) survey, developed by the US Agency for Healthcare Research and Quality (AHRQ) to measure hospital-based inpatient satisfaction [1]. However, patient satisfaction metrics have expanded into the outpatient setting, and the Press Ganey® Medical Practice survey has become the most commonly used survey of outpatient satisfaction in the United States [5]. The Press Ganey® survey is used by health care administrations as a metric assessing various aspects of health care delivery such as wait-times in clinic and the patient-provider interaction. At our institution, which is comprised of hospitals and clinics that provide primary through tertiary care for over 200 medical specialties; the Press Ganey® survey’s provider level summaries are publicly available online to guide prospective patients [6]. Systems of compensation at the provider level vary within our institution, ranging from salaried to collections-based. It is important for the Press Ganey® survey to be reliable and valid given that results from these surveys may impact patronage, are being factored into reimbursement models, and currently influence policy decisions.

Psychometric analyses of the H-CAHPS inpatient and the outpatient Clinician and Group CAHPS (CG-CAHPS) surveys are available in the literature, and both surveys have generally been found to have good reliability and validity [7–12]. Differences between the H-CAHPS and CG-CAHPS (ie “CAHPS surveys”) and the Press Ganey® include the following: a) the CAHPS surveys use a measure patient satisfaction with the medical care received over the past year within b) a random sample of the patient population who are contacted by c) mail, telephone, email (with mail or telephone) or mixed mode protocols [13]. In contrast, the Press Ganey® survey is a modified version of the CG-CAHPS designed to evaluate patient satisfaction with outpatient care received at every visit (ie, at the encounter level), and at our institution it is sent exclusively via e-mail to all patients following all outpatient visits. A psychometrics report for the Press Ganey® survey is available from the vendor, providing assessments of reliability and validity based on 1791 surveys from 8 practices across 5 states [14]. This report describes a 34 question version of the survey with “no problems encountered” with missingness and response variability (page 2, [14]). The report further shows the Press Ganey® survey to demonstrate construct, convergent, divergent and predictive validities, and high reliability. Despite its widespread use and impact, to our knowledge there is no peer-reviewed literature on the psychometric properties of the Press Ganey® survey.

Given the increasing impact of patient satisfaction data on the way that health care is delivered, compared, and funded, there is a need for validation of the Press Ganey® survey by independent investigators. Here we evaluate the psychometric properties of the Press Ganey® survey from annual outpatient encounters in a single institution setting.

## Methods

### Sample and data

Approval for this study was obtained from our Institutional Review Board. The raw dataset consisted of 62,801 Press Ganey® Medical Practice surveys from 34,534 patients receiving care from 664 providers between 1/1/2013 and 12/31/2013 from our institution, which is a University-based health care system comprised of hospitals and clinics that provide primary through tertiary care for over 200 medical specialties. Patients were sent an email with a link to the Press Ganey® survey following their visit. Surveys missing all 24 items were excluded. If a patient filled out multiple surveys in 2013, only the first survey with at least 1 of the 24 items answered was kept. The final analysis data set contained 34,503 surveys from unique patients seen by 624 providers from 47 specialties and 94 clinics.

The Press Ganey® survey has 24 items organized into 6 scales: Access (4 items), Moving Through the Visit (2), Nurse Assistant (2), Care Provider (10), Personal Issues (4) and Overall Assessment (2). Each item was scored as follows: very poor (score = 0), poor (25), fair (50), good (75) and very good (100). The score for each scale was calculated from the mean scores of all items within the scale, and the mean total score was calculated from the mean scores from the six scales weighted equally. The Press Ganey® survey scoring instructions document is available by contacting Press Ganey® [15]. Consistent with factor analyses performed on the CAHPS surveys, confirmatory factor analysis was performed on the first 22 questions composing the first five scales [7–9, 11, 12], as the sixth scale corresponded to Overall Assessment (two questions), which correlated with the other scales.

### Psychometric methods

We sought to evaluate both the reliability and validity of the Press Ganey® survey to assess its utility for measuring patient satisfaction. Reliability is the extent to which a survey measures true signal. Validity indicates the extent to which a survey measures what it is intended to measure. In particular we evaluated the following Press Ganey® survey properties: 1) data quality, 2) internal consistency reliability of items within each scale, 3) factor structure, 4) convergent validity and 5) discriminant validity.

Items, scales and total scores were evaluated for missingness, the percentage of values hitting the floor (minimum value) and ceiling (maximum value), and skewness. To assess whether items were missing completely at random, we used Little’s MCAR test implemented in the BaylorEdPsych package in R [16]. High floor and ceiling rates yield reduced power to discriminate among patients who have low or high satisfaction, respectively. Floor and ceiling rates were defined as rare if they occurred < 5% of the time and substantial if they occurred >20% of the time [17, 18]. These data quality metrics can impact the reliability and validity of a survey [17, 18]. In particular, substantial ceiling (or floor) rates can notably impact percentile rankings of scores within an institution. To investigate this we converted the raw scores to percentile ranks using two different methods: method 1) the empirical cumulative distribution function in R ecdf(), and method 2) dividing the rank of a score by the number of scores. To assess how a provider’s percentile rank score could change quarterly, we calculated percentile rank scores within each quarter, and then averaged the raw and percentile rank scores for each provider by quarter. We then summarized the median change in consecutive quarters for raw and percentile rank scores among a) all providers, and b) providers who had a perfect score in at least one quarter.

We analyzed internal consistency reliability and homogeneity of items within each scale using Cronbach’s alpha and inter-item correlations [19, 20]. Nunnally recommends a minimum of 0.7 for Cronbach’s alpha [21]. Briggs and Cheek [22] suggested mean interitem correlations in the range of 0.1–0.5, and Clark and Watson [23] encouraged all interitem correlations to fall within this range. Smaller values (<0.1) suggest too much variability for the items to represent a single scale, and larger values (>0.5) suggest item redundancy. We also calculated item-scale correlations, corrected for item overlap, where a 0.4 benchmark supported internal consistency [21]. The goal of these analyses was to verify consistency of items within scales, which is important for yielding reproducible results.

We explored the factor structure of the Press Ganey® survey numerically using confirmatory factor analysis (CFA) and visually using multi-dimensional scaling (MDS). The MDS plot provided a tool to visualize how items clustered within scales and inter-relationships among the scales based on Euclidean distances between the items. CFA was used to test whether the data fit the Press Ganey® survey design using the fit indices: root mean square error of approximation (RMSEA, cut off for good fit <0.06), comparative fit index (CFI, >0.95), Tucker-Lewis index (TLI, >0.9), and standardized root mean square residual (SRMR, <0.08) [24].

CFA was also used to evaluate the convergent validity of the survey, whether items within a scale were similar, by evaluating whether the average variance extracted (AVE) of each construct was greater than 0.5 [12, 25]. We also evaluated convergent validity by correlating the items and scales with the overall assessment questions. Correlations that were statistically significant supported the convergent validity of the Press Ganey® survey [8]. CFA was also used to evaluate discriminant validity, item uniqueness and the extent with which an item relates more to its own scale than other scales, by checking that each AVE was greater than the squared correlations with the other constructs [25].

CFA parameters were estimated using a robust weighted least squares estimator, which is the default for ordered categorical variables in Mplus v. 7.0 software. This approach used probit regression to model ordered indicators for each of the 22 items as linear combinations of latent factors. In addition to single-level CFA, we also evaluated the extent of clustering within providers and clinics; ie “clusters”; using the intraclass correlation coefficient (ICC) and the design effect. The ICC indicates similarity of satisfaction scores from patients within clusters, and is calculated as: between cluster variability/(within cluster variability + between cluster variability). Higher ICC values indicate greater similarity of scores within clusters. The design effect adjusts the ICC for the average cluster size, and thus gives a better idea of the impact of clustering on the analysis. A higher design effect indicates a greater loss of statistical power due to within cluster similarity, and emphasizes that clustering should be accounted for in the analysis. Multi-level CFA was used to confirm discriminant and convergent validity results from the single-level CFA. Statistical analyses were conducted in Mplus v. 7.0 with the multi-level add-on and R v. 3.03.

## Results

According to Lee et al. [6], the response rate at our institution for the Press Ganey® survey in both fiscal years 2013 and 2014 was 12.6%, which is a close approximation to the rate in our study [6]. While individual item missingness was generally low, ranging from 0.8 to 11.4%, 13.4% of surveys had ≥2 missing items, 2.7% had ≥5, and 0.02% of surveys were scored with only a single question answered. Missingness differed across items (Little’s MCAR *p* < 0.001), where items with highest missingness included questions asking patients to rate information about medications (11.4% missing) and the degree to which patients were informed of delays (8.6% missing).

Ceiling rates were high, with items hitting maximum values 55.4–84.1% of the time, scales hitting maximum values 45.6–75.6% of the time, and total scores (calculated from all 24 items) hitting the maximum value 29.3% of the time (Table 1). Floor effects were low, with items, scales and total scores hitting the minimum value < 2.5% of the time. Skewness ranged from −3.5 to −1.3 across items.

**Table 1:** Data quality, internal consistency reliability, construct validity

					Correlations^a^		
	%Floor	%Ceiling	Skew	%NA	Scale	PG23	PG24
Access, α = 0.79	0.05%	45.6%	−1.3	0.5%	0.49	0.51	0.46
PG1 Ease of getting clinic on the phone	0.70%	57.0%	−1.6	3%	0.62	0.36	0.32
PG2 Convenience of office hours	0.20%	59.1%	−1.3	1.10%	0.64	0.40	0.36
PG3 Ease of scheduling appointment	0.70%	65.1%	−1.9	1.10%	0.66	0.40	0.37
PG4 Courtesy of staff in registration area	0.20%	78.5%	−2.5	1.40%	0.49	0.46	0.41
Moving through visit, α = 0.88	0.98%	49.9%	−1.6	1.62%	0.75	0.50	0.45
PG5 Informed of any delays	1.80%	57.9%	−1.7	8.60%	0.75	0.49	0.43
PG6 Wait time at clinic	2.10%	55.4%	−1.5	2.30%	0.75	0.46	0.42
Nurse/Assistant, α = 0.89	0.13%	73.0%	−2.3	1.48%	0.82	0.60	0.51
PG7 Friendliness of nurse	0.10%	79.6%	−2.5	1.70%	0.82	0.56	0.47
PG8 Concern nurse showed for problem	0.30%	73.9%	−2.2	2.60%	0.82	0.58	0.50
Care Provider (CP), α = 0.96	0.12%	64.4%	−3.0	0.55%	0.75	0.69	0.77
PG9 Friendliness of CP	0.30%	84.1%	−3.3	0.80%	0.82	0.61	0.65
PG10 Explanations provided for problem	0.50%	80.4%	−2.9	1.10%	0.88	0.61	0.66
PG11 Concern for your questions	0.70%	80.4%	−3.1	1.30%	0.90	0.62	0.69
PG12 Efforts to include you in treatment	0.70%	79.6%	−3.0	2.10%	0.88	0.61	0.67
PG13 Info. about medications (if any)	0.60%	77.2%	−2.7	11.40%	0.84	0.60	0.63
PG14 Instructions for care (if any)	0.60%	76.7%	−2.6	5.60%	0.83	0.61	0.64
PG15 Understandable explanations	0.30%	83.3%	−3.3	1.50%	0.81	0.59	0.61
PG16 Time spent with you	0.60%	76.3%	−2.6	1.20%	0.80	0.60	0.63
PG17 Your confidence in CP	0.80%	83.2%	−3.5	1.30%	0.88	0.62	0.75
PG18 Likelihood of recommending CP	1.30%	83.0%	−3.4	1.90%	0.86	0.63	0.80
Personal Issues, α = 0.89	0.06%	67.6%	−2.0	1.06%	0.68	0.73	0.68
PG19 Staff protected your safety	0.20%	75.0%	−2.2	4.70%	0.74	0.57	0.50
PG20 Our sensitivity to your needs	0.50%	75.0%	−2.6	2.20%	0.76	0.73	0.71
PG21 Our concern for your privacy	0.20%	76.7%	−2.3	3%	0.81	0.64	0.58
PG22 Cleanliness of our practice	0.10%	80.1%	−2.3	2.60%	0.75	0.59	0.55
Total Score, α = 0.95	0.01%	29.3%	−1.7	0.00%	--	--	--

^a^Scale refers to correlation of each item with its scale, adjusted for removing the current item. Since the correlation of a scale with itself is 1, the average inter-item correlations within the scale is provided. PG23 is “How we took care of you”, and PG24 is “Likelihood of recommending” from the overall assessment domain

To study the consequence of the high ceiling rate in our data set, we converted raw scores to percentile scores and identified the two highest raw scores in our data set: 100 and 99.5. A perfect raw score of 100 corresponded to the 100^th^ or 85.4^th^ percentile, depending on the percentile rank method used (methods 1 or 2, respectively); and a raw score of 99.5 corresponded to the 70.7^th^ and 70.4^th^ percentile respectively. Thus, if a provider received a perfect score in quarter 1, but then experienced a 0.5 point decrease in his/her raw score in quarter 2, this could correspond to a 29.3 or 15 point decrease in percentile rank score depending on the percentile rank method used. Next we examined how the ceiling rate typically affected provider percentile scores across consecutive quarters at our institution. Comparing the providers with a perfect score for at least one quarter with the providers without a perfect score, are helpful for illustrating the impact of the ceiling effect on percentile score changes across quarters. Among providers who had a perfect score in at least one quarter, the median change was 6.5 points for the raw score and 33.9 or 26.1 points for the percentile score (by methods 1 and 2, respectively). In contrast, providers who did not enjoy a perfect score had a median change of 3.1 points for the raw score and 9.2 or 8.0 points for the percentile score. This shows that the ceiling effect leads to a 3.3–3.7 fold change in the quarterly percentile scores.

### Internal consistency reliability

Internal consistency reliability was high for all scales, with values ranging from α = 0.79–0.96, exceeding the 0.7 benchmark (Table 1) [21]. The Access scale had the lowest internal consistency reliability at 0.79, and the Care Provider scale had the highest at 0.96. Items within a scale had correlations with the scale ranging from *r* = 0.49–0.9, exceeding the 0.4 benchmark supporting internal consistency [21].

Interitem correlations within scales ranged from 0.38 (PG4 vs.PG1) to 0.85 (PG10 vs. PG11), where all but three inter-item correlations exceeded the 0.1–0.5 ideal range, suggesting potential item redundancy. In particular, the 10-item Care Provider scale had interitem correlations ranging from 0.69 to 0.91, where “PG10 Explanations provided for problem”, “PG11 Concern for your questions”, and “PG12 Efforts to include you in treatment” had pair-wise correlations ranging from 0.82 to 0.85; and “PG17 Your confidence in CP” and “PG18 Likelihood of recommending CP” had a correlation of 0.91.

### Factor structure analysis and evaluation of convergent and discriminant validities

**Fig. 1:**
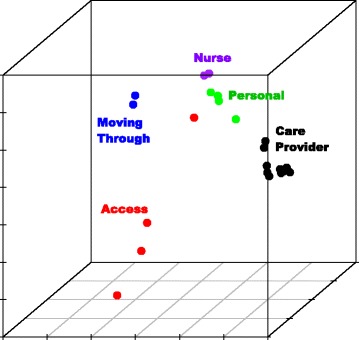
Multi-dimensional scaling plot of the Press Ganey® survey questions colored by scale

CFA supported the 5-factor model, where all fit index criteria were met: RMSEA = 0.051, CFI = 0.99, TLI = 0.99, SRMR = 0.02. The MDS plot supported the 5-factor structure and showed that all items within a scale were fairly clustered except for “PG4 Courtesy of staff in registration area” from the Access scale (Fig. 1). This item had the lowest inter-item correlation within the Access scale (0.38–0.44), although these correlations were within the desirable range.

CFA supported the convergent validity of the Press Ganey® survey because the AVE exceeded 0.5 for all constructs (Table 2). It also supported the discriminant validity of the survey by the AVE exceeding the squared correlation of the other constructs. Correlation between the scales and the overall assessment questions “PG23 How we took care of you” and “PG24 Likelihood of recommending” also indicated high convergent validity with correlations ranging from 0.50 to 0.73 and 0.45–0.77, respectively. All correlations were statistically significant.

**Table 2:** Factor loadings and estimated correlation matrix

Domain	Item	Loading	Access	Moving through	Nurse	Provider	Personal
Access, AVE: 0.68	PG1	0.77	1	--	--	--	--
	PG2	0.84					
	PG3	0.84					
	PG4	0.86					
Move through, AVE: 0.84	PG5	0.96	0.67	1	--	--	--
	PG6	0.88					
Nurse, AVE: 0.93	PG7	0.96	0.71	0.64	1	--	--
	PG8	0.98					
	PG9	0.94					
	PG10	0.95					
Provider, AVE: 0.89	PG11	0.97	0.66	0.63	0.70	1	--
	PG12	0.96					
	PG13	0.94					
	PG14	0.93					
	PG15	0.93					
	PG16	0.91					
	PG17	0.95					
	PG18	0.95					
Personal, AVE: 0.84	PG19	0.87	0.77	0.70	0.79	0.85	1
	PG20	0.97					
	PG21	0.93					
	PG22	0.89					

We confirmed the CFA results using a multi-level CFA that accounted for clustering within clinic and provider. There were 624 care providers (averaging 55 patients/provider, ranging from 1 to 409 patients/provider) and 94 clinics (averaging 367 patients/clinic, ranging from 1 to 3424 patients/clinic). The ICC was higher for provider (ICC = 0.06) than clinic (ICC = 0.03), suggesting that scores within a provider were more similar than scores within a clinic. However, since the average cluster size for clinics was larger, the design effect was higher for clinics (design effect clinic = 11.98, design effect provider = 4.24). The multi-level CFA for clinic also met all fit criteria for the 5-factor model: RMSEA = 0.013, CFI > 0.99, TLI > 0.99, SRMR within = 0.03, SRMR between = 0.05. It yielded similar results for the AVE estimates, supporting the convergent and discriminant validity of the survey. The multi-level CFA for provider yielded similar results.

## Discussion

Our single institution psychometric analysis of the Press Ganey® Medical Practice survey for measuring outpatient satisfaction found desirable reliability and validity. Consistent with other studies, we observed negatively skewed distributions for all items, with a ceiling rate of 29.3% for the Press Ganey® survey total score [7, 8]. Our item-level ceiling rates were 55.4–84.1%, consistent with ceiling rates of 25.1–76% reported by Arah et al. [7] for the Dutch version of the H-CAHPS, and 40–95% reported by Dyer et al. [8] for the CG-CAHPS. The high ceiling rate for the Press Ganey® survey total score is a limitation given that total scores are translated into percentile rank scores which are used to assess institution and provider performance. Press Ganey® provides percentile rank scores based on a comparison of raw scores among all participating US hospitals. In our data set the ceiling rate indicated that if percentile ranks were calculated within our institution, a provider’s average score could potentially drop from the 100^th^ to 70.7^th^ with the loss of a half point in the raw score (from 100 to 99.5). This is especially problematic for a provider who sees few patients in a quarter, where a shift from a perfect score is more likely. We are unsure of whether percentile rank scores calculated within our institution are used to asses provider or department performance. However, given the consistently high ceiling rates published for patient satisfaction, it is conceivable that Press Ganey®’s percentile rank scores also vary substantially from one measurement time period to the next.

Relative to CAHPS response rates, which are typically 30–40%, our 12.6% response rate is somewhat low [26, 27]. However, there are differences in how the CAHPS and Press Ganey® survey are administered. The CAHPS are administered annually or semi-annually to a randomly selected set of patients with a goal of achieving 300 completed surveys using mail, telephone, email (with mail or telephone) or mixed mode protocols [13]. At our institution, the Press Ganey® survey is administered to all patients via email following every encounter in our health system, so the lower rate is potentially due to response fatigue. The low response rate suggests potential for non-response bias -- our responders may not represent the typical patient population but rather correspond to a subset who are more or less satisfied. However, this is only an issue if respondent characteristics differ over time or across hospitals. Given that Press Ganey® survey responses can be linked to encounters and thus patient characteristics, it would be helpful to monitor patient characteristics of responders and non-responders over time to aid in interpretation of temporal trends. Also, adjustment for patient characteristics (case-mix), similarly to what is done for CAHPS, would be helpful for comparing satisfaction scores across institutions [28]. Currently, Press Ganey® adjusts for survey modality but not case-mix [29]. The value of case-mix adjustment is two-fold, it would enable greater comparability of patients across hospitals, and it would mitigate the effects of non-response bias [26].

Another potential issue with data quality could be the scoring method, as currently a survey will be scored if only a single question has been answered. Thus, a scored survey that has many missing values may have questionable validity and reliability [17, 18]. While we did not find this to be an issue in our data set, where 13.4% had two or more missing values and only 0.02% of surveys were scored based on a single item, it is a potential limitation. Data quality checks such as missingness could be conducted prior to using scores for performance evaluation or reimbursement purposes.

Consistent with the psychometric report published by Press Ganey®, we also found high reliability, convergent validity and divergent validity. Cronbach’s alpha estimates ranged from 0.79 to 96 across the five scales, which were similar to the psychometric report’s range of 0.81–0.97. Our results were also consistent with the psychometric findings from the CG-CAHPS and H-CAHPS, where most have reported acceptable reliability and validity [7–10].

Assurance of the reliability and validity of the Press Ganey® survey is important as patient satisfaction is increasingly factored into administrative decision making and reimbursement. To our knowledge, this single institutional study was the first to evaluate the psychometric properties of the Press Ganey® survey. A follow-up study of the Press Ganey® survey’s psychometric properties across a nation-wide representative sample of institutions is needed to support its use in evaluating national performance of providers and institutions.

## Conclusions

Our single institutional psychometric analysis of the Press Ganey® Medical Practice survey for measuring outpatient satisfaction found acceptable reliability and validity. While high ceiling rates are typical for patient satisfaction surveys, we found that 29.3% of surveys achieved the maximum score. As a result, provider percentile rank scores within our institution could change by 29.3% with a half point decrease from a perfect raw score. The 12.6% response rate suggests potential for non-response bias. Case-mix adjustment would mitigate the effects of potential non-response bias and improve score comparability across time and institutions. Further studies are needed to investigate the psychometric properties of this survey at a multi-institutional level.

## Abbreviations

AHRQ: US agency for healthcare research and quality
AVE: Average variance extracted
CAHPS: Clinician and group consumer assessment of healthcare providers and systems
CFA: Confirmatory factor analysis
CFI: Comparative fit index
CG-CAHPS: Outpatient clinician and group consumer assessment of healthcare providers and systems
H-CAHPS: Hospital consumer assessment of healthcare providers and systems
ICC: Intraclass correlation coefficient
MDS: Multi-dimensional scaling
Press Ganey® survey: Press Ganey® outpatient medical practice survey
RMSEA: Root mean square error of approximation
SRMR: Standardized root mean square residual
TLI: Tucker-Lewis index
